# Supplementary material for: Endophilin A2 regulates B‐cell endocytosis and is required for germinal center and humoral responses
Source: EMBO Rep. 2021 Jul 29;22(9):e51328. doi: 10.15252/embr.202051328 (PMC8419706; doi:10.15252/embr.202051328)
Supplement: Supplementary file 2 — Expanded View Figures PDF [file EMBR-22-e51328-s004.pdf]

Expanded View Figures

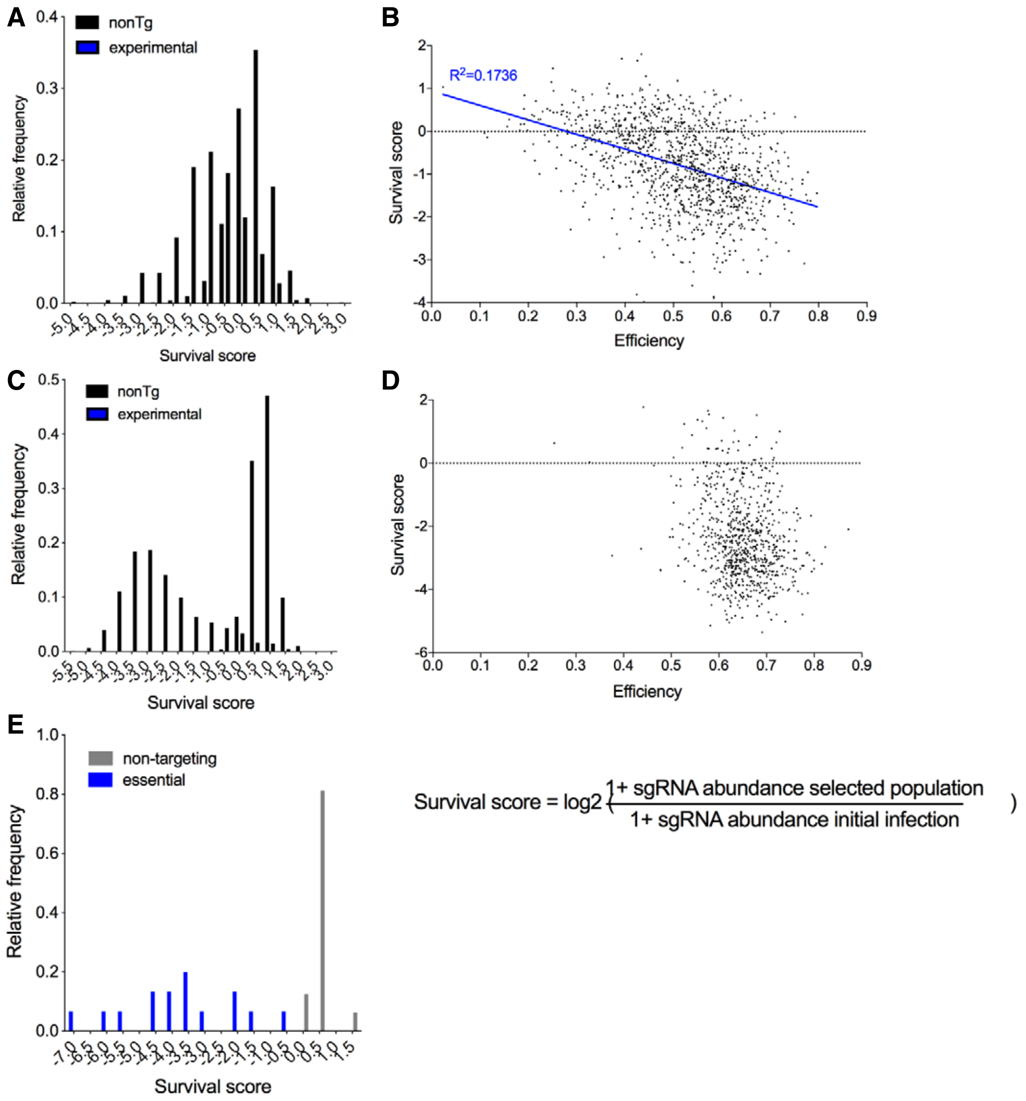

Figure EV1. Analysis of essential gene targeting in large-scale pooled CRISPR screens.

A GeCKO library essential gene survival scores. The plot shows mean CRISPR survival scores of sgRNAs targeting a set of top 200 essential genes (Wang *et al*, 2015).
B Correlation of the CRISPR survival score and sgRNA on-target efficiency for a set of sgRNAs targeting the essential genes from the GeCKO library. On-target efficiency was calculated using "Rule Set 2" algorithm (Doench *et al*, 2016).
C Brunello library essential gene survival scores.
D Correlation of survival score and sgRNA on-target efficiency in Brunello library.
E Custom minilibrary essential gene survival scores.

© 2021 The Authors

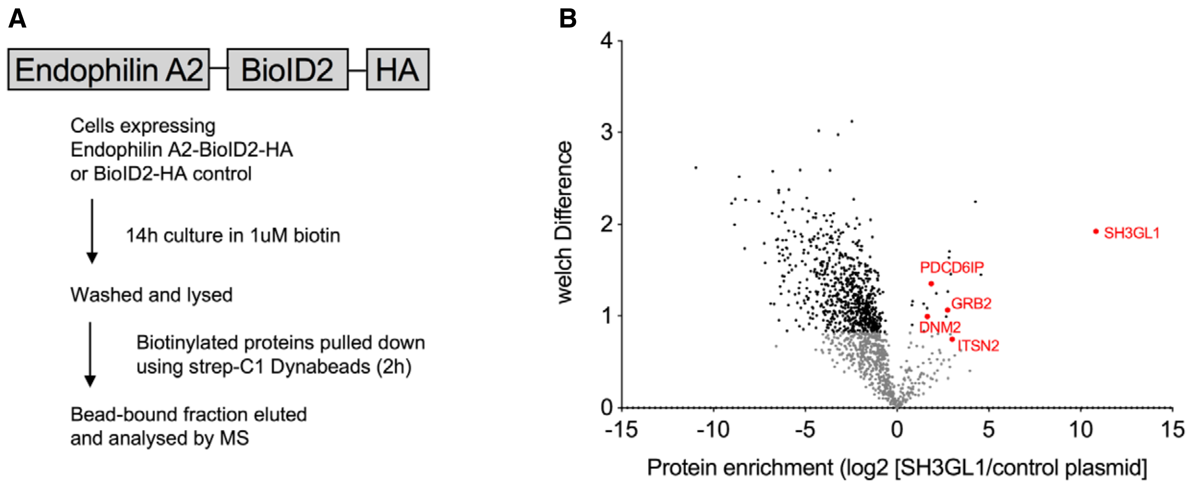

Figure EV2. BioID2 reveals potential endophilin A2 binding partners.

A BioID2-tagged endophilin A2 construct and assay workflow.

B Proteins significantly enriched in biotinylated fraction of endophilin A2-BioID2 samples compared with empty plasmid control (positive protein enrichment scores). Negative enrichment score represents gene products enriched in BioID2 control sample, reflecting the promiscuous nature of the cytoplasmic biotinylase. Data from 2 independent experiments, see also Dataset EV4.

© 2021 The Authors

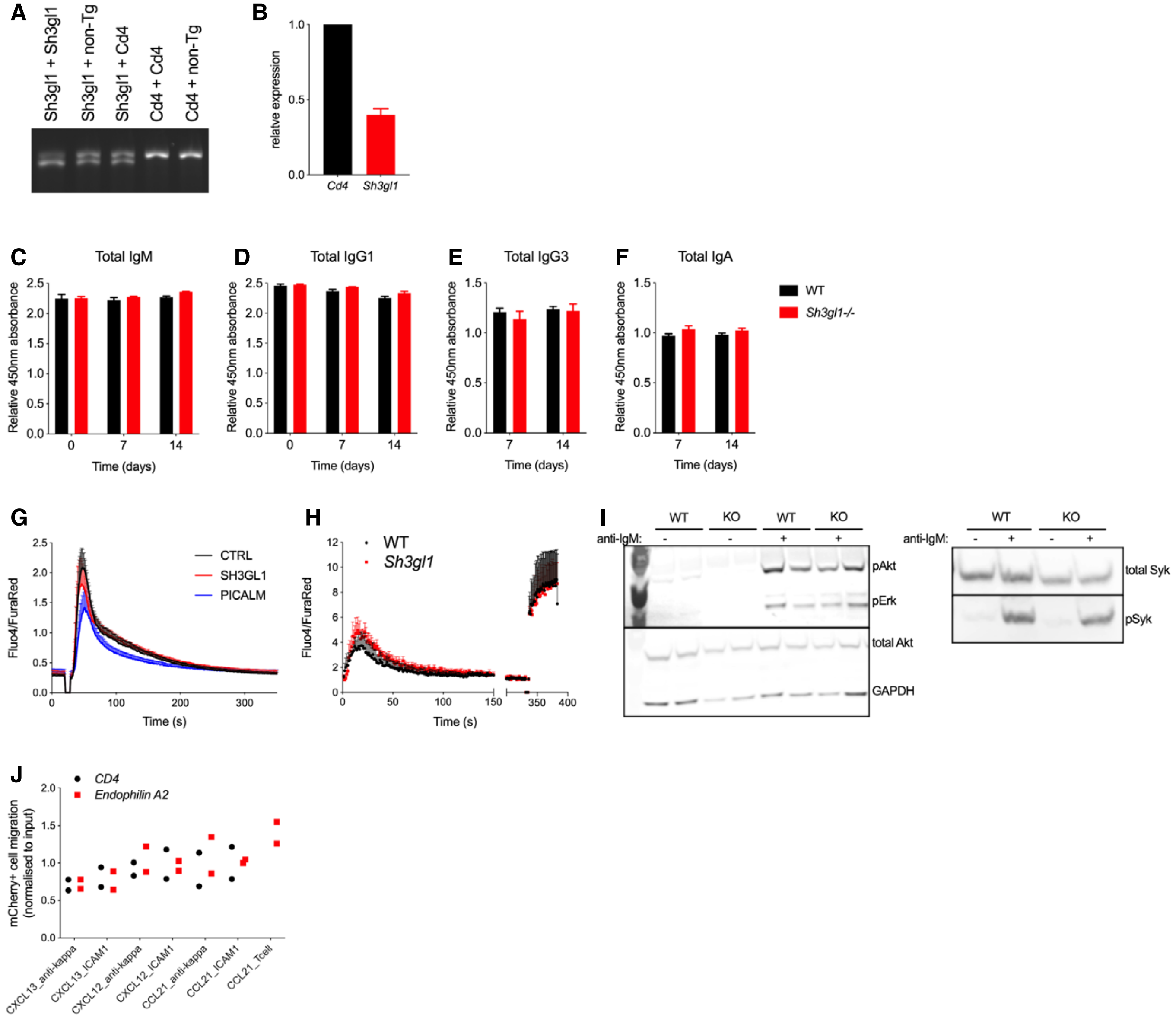

Figure EV3. *Sh3gl1* deletion does not affect total antibody titers or early BCR signaling.

A Surveyor assay detecting genomic DNA editing. 200bp genomic region around the targeted PAM site in *Sh3gl1* is amplified from cells expressing *Sh3gl1*, *Cd4*, or non-targeting sgRNA. PCR amplicons from targeted and wild-type DNA are mixed, denatured, and re-annealed. CRISPR-mediated editing results in mis-matched DNA loops, which are cleaved by an endonuclease and visualized as two separate bands.

B *Sh3gl1* transcript levels in sorted donor B-cell populations from chimeric mice with mCherry expression between 40 and 60%. Data show mean + SEM from four mice in two experiments.

C–F Total antibody levels measured by clonotyping ELISA using capture antibody. One representative experiment showing measurements from 4 mice. Data show mean + SEM. (C) IgM (D) IgG1 (E) IgG3 (F) IgA.

G CRISPR-targeted Ramos, activated with 2 μg/ml of anti-human IgM F(ab')$_2$. Data show mean and SEM from 4 independent infections.

H Primary B cells from *Sh3gl1*$^{-/-}$ mice or WT littermates, activated with 2 μg/ml anti-mouse Igκ F(ab')$_2$, for 380 seconds, followed by addition of ionomycin (5 μg/ml) at 300 s. $N = 3$ mice. Data show mean + SEM.

I Western blot detection of total and phosphorylated Syk, Erk, and Akt in primary B cells from *Sh3gl1*$^{-/-}$ mice or WT littermates, activated with 2 μg/ml anti-mouse Igκ F(ab')$_2$.

J Chemokine-induced migration through ICAM-1 or anti-Igκ-coated transwells. mCherry$^+$ percentage is normalized to input population. $N = 2$ mice.

 © 2021 The Authors

Figure EV4. Loss of endophilin A2 affects cell expansion in primary and Ramos B cells.

A Heat map of TPM values from RNAseq comparing follicular B cells from WT and *Sh3gl1*$^{-/-}$ littermates. Shown are the top 20 genes upregulated in WT B cells 24 h after CD40L activation.

B Numbers of viable cells in 3-day culture of follicular B cells in specified cytokines. $N = 3$ mice. Data show mean and SEM; *P* values are calculated using two-way ANOVA.

C AnnexinV stain of B-cell cultures in (B). Data show mean and SEM; *P* values are calculated using two-way ANOVA.

D Endophilin A2, EPN1, and PICALM survival scores in hematopoietic cell lines from our screens using the Brunello library in Ramos cells, together with CRISPR scores from published genome-wide screens (Wang *et al*, 2015; Wang *et al*, 2017; Phelan *et al*, 2018). Scores are normalized using the mean value of the top 200 essential genes in each screen. Dotted line indicates typical CRISPR score threshold for gene essentiality.

E Decreased percentage of *SH3GL1*-targeted Ramos population in experiments with indicated initial percentage of targeted cells. Data show one representative experiment out of 3.

F BrdU pulse-chase assay in Ramos cells. Clockwise from top left: equal BrdU incorporation at time = 0 h; slower progression of BrdU-labeled cells into G2 phase in *SH3GL1*- compared with CTRL-targeted cells at time = 4 h; slower progression into G1 of following cycle while greater proportion of *SH3GL1* cells remain in initial S phase at time = 8 h; slower progression between G2 and M phase in *SH3GL1*-targeted cells resulting in significantly greater numbers in G2 and less in G1 or S phase of subsequent cycle. $N = 4$ independent infections. Data show mean + SEM. $^{*}P \leq 0.05$ $^{**}P \leq 0.01$ $^{***}P \leq 0.001$ $^{****}P \leq 0.0001$ using 2-way ANOVA with multiple comparisons.

G GSEA of RNAseq analysis comparing WT and *Sh3gl1*$^{-/-}$ sorted follicular B cells. Three mice of each genotype were used for RNAseq and top 10 significantly enriched gene sets up- or down-regulated in *Sh3gl1*$^{-/-}$ B cells are shown.

H Expression of genes upregulated in *Sh3gl1*$^{-/-}$ B cells in GO: 0006879 cellular_iron_ion_homeostasis. $N = 3$ mice. Data show mean ± SEM.

© 2021 The Authors

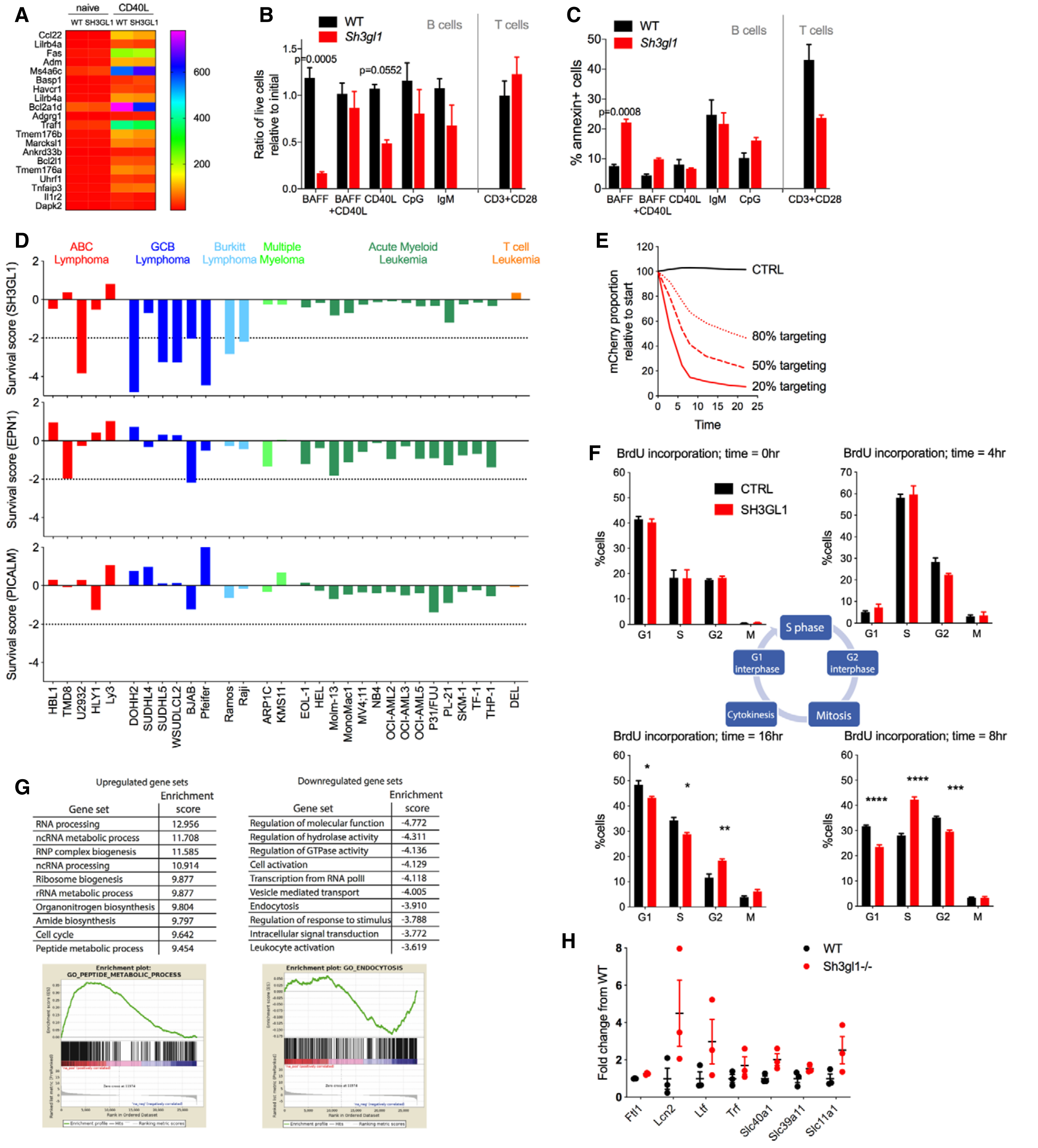

Figure EV4.

 © 2021 The Authors

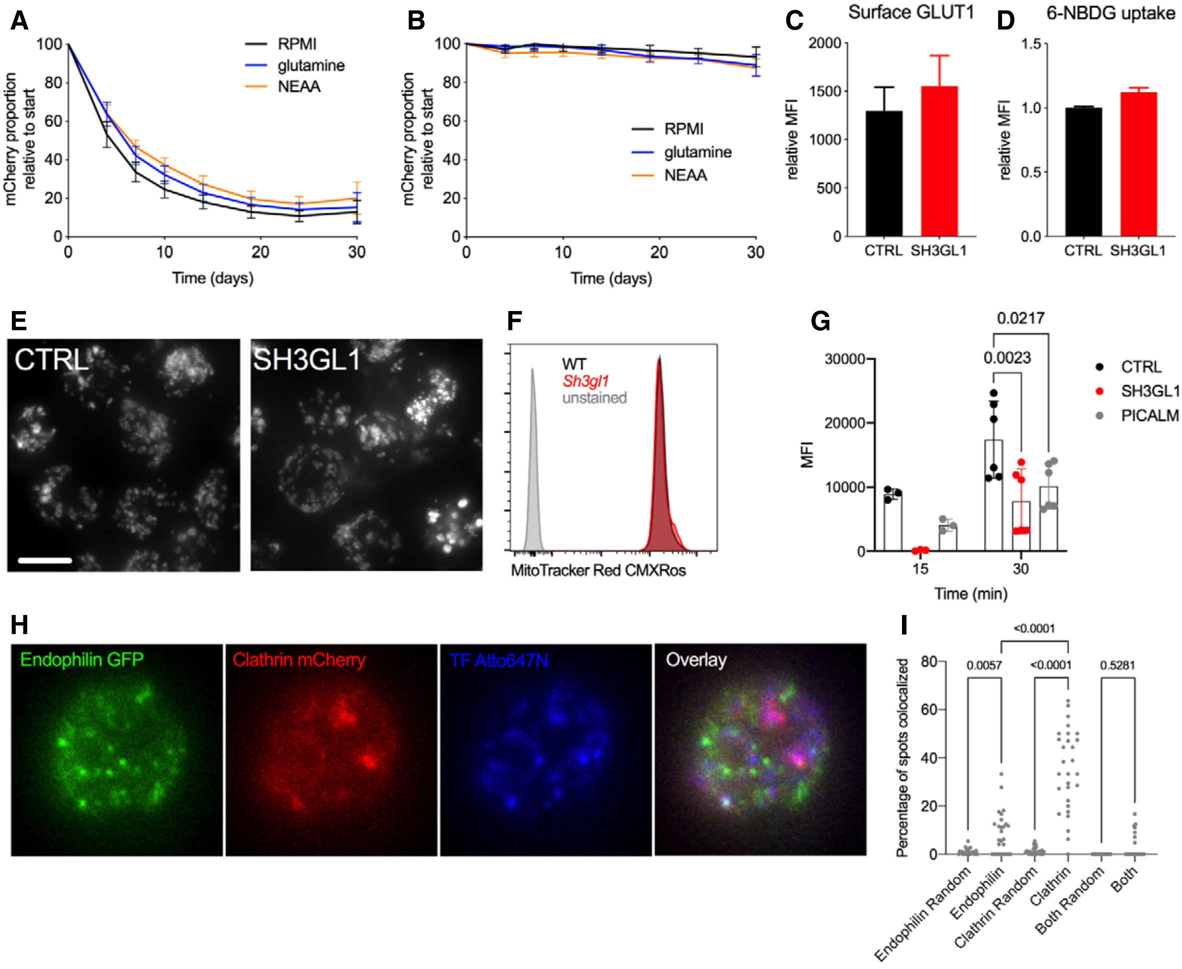

Figure EV5. Growth defect upon *SH3GL1* deletion is not rescued by common cell culture supplements and does not correlate with altered mitochondrial mass.

A mCherry percentage in *SH3GL1*-targeted Ramos cells over time, supplemented with 5× normal concentration of L-Glutamine or NEAA. *N* = 5 independent infections. Data show mean ± SEM.

B mCherry percentage in CTRL-targeted Ramos cells over time, supplemented with 5× normal concentration of L-Glutamine or NEAA. *N* = 5 independent infections. Data show mean ± SEM.

C Surface GLUT1 stain in CRISPR-targeted Ramos cells, *N* = 8 across 3 experiments. Data show mean ± SEM.

D Accumulation of 6-NBDG (fluorescent glucose analog) after 10-min internalization in Ramos cells; *N* = 4. Data show mean ± SEM.

E Anti-TUFM immunofluorescence showing total mitochondrial mass and organization in CRISPR-targeted Ramos cells. Scale bar = 5 μm.

F MitoTracker Red CMXRos stain for active mitochondria in primary B cells from WT and $Sh3gl1^{-/-}$ B cells.

G Human transferrin uptake in targeted Ramos cells. Data show mean ± SEM from six independent CRISPR infections in two experiments; significance calculated using two-way ANOVA.

H Ramos expressing clathrinLC-mCherry and endophilinA2-GFP internalize Atto647N-labeled human transferrin.

I Quantification of transferrin clusters with either clathrin spots, endophilinA2 spots, or both. *N* = 25–30 cells across two experiments. Significance calculated using two-way ANOVA.

© 2021 The Authors
