# Supplementary material for: Endophilin A2 regulates B‐cell endocytosis and is required for germinal center and humoral responses
Source: EMBO Rep. 2021 Jul 29;22(9):e51328. doi: 10.15252/embr.202051328 (PMC8419706; doi:10.15252/embr.202051328)
Supplement: Supplementary file 8 — Movie EV1 [file EMBR-22-e51328-s007.zip › Movie/MovieEV1.rtf]

Movie EV1. Live TIRF imaging of a Ramos cell expressing endophilin A2-GFP (green) and mCherry-Clathrin LC (magenta). The cell is interacting with anti-IgM F(ab’)2 -loaded PMS (blue). Simple ratio bleach correction (Fiji) was performed on the antigen channel. Yellow boxes indicate instances of endophilin A2-GFP recruitment to antigen clusters in the absence of clathrin LC signal. Scale bar = 5 μ.
